# Resuscitative Endovascular Balloon Occlusion of the Aorta: Review of the Literature and Applications to Veterinary Emergency and Critical Care

**DOI:** 10.3389/fvets.2019.00197

**Published:** 2019-06-19

**Authors:** Guillaume L. Hoareau, Emily M. Tibbits, Carl A. Beyer, Meryl A. Simon, Erik S. DeSoucy, E. Robert Faulconer, Lucas P. Neff, J. Kevin Grayson, Ian J. Stewart, Timothy K. Williams, M. Austin Johnson

**Affiliations:** ^1^Clinical Investigation Facility, David Grant USAF Medical Center, Travis Air Force Base, Travis, CA, United States; ^2^Department of Surgery, University of California Davis Medical Center, Sacramento, CA, United States; ^3^Vascular Surgery, Derriford Hospital, Plymouth, United Kingdom; ^4^Department of Surgery, Wake Forest Baptist Medical Center, Winston-Salem, NC, United States; ^5^Department of Medicine, Uniformed Services University of the Health Sciences, Bethesda, MD, United States; ^6^Department of Vascular and Endovascular Surgery, Wake Forest Baptist Medical Center, Winston-Salem, NC, United States; ^7^Department of Emergency Medicine, University of California Davis Medical Center, Sacramento, CA, United States

**Keywords:** hemorrhage, trauma, non-compressible truncal hemorrhage, endovascular trauma management, shock

## Abstract

While hemorrhagic shock might be the result of various conditions, hemorrhage control and resuscitation are the corner stone of patient management. Hemorrhage control can prove challenging in both the acute care and surgical settings, especially in the abdomen, where no direct pressure can be applied onto the source of bleeding. Resuscitative endovascular balloon occlusion of the aorta (REBOA) has emerged as a promising replacement to resuscitative thoracotomy (RT) for the management of non-compressible torso hemorrhage in human trauma patients. By inflating a balloon at specific levels (or zones) of the aorta to interrupt blood flow, hemorrhage below the level of the balloon can be controlled. While REBOA allows for hemorrhage control and augmentation of blood pressure cranial to the balloon, it also exposes caudal tissue beds to ischemia and the whole body to reperfusion injury. We aim to introduce the advantages of REBOA while reviewing known limitations. This review outlines a step-by-step approach to REBOA implementation, and discusses common challenges observed both in human patients and during translational large animal studies. Currently accepted and debated indications for REBOA in humans are discussed. Finally, we review possible applications for veterinary patients and how REBOA has the potential to be translated into clinical veterinary practice.

## Introduction

Resuscitative endovascular balloon occlusion of the aorta (REBOA) is an endovascular hemorrhage control intervention that was first described in the Korean war (1). REBOA has recently re-gained popularity as an alternative to resuscitative thoracotomy (RT, thoracotomy and aortic cross-clamping) in trauma patients (2–8). RT has been associated with low survival rates (9, 10) and presents significant risks of injuries for care providers themselves. Furthermore, while RT may provide hemorrhage control, the thoracotomy itself adds a substantial injury to the patient with considerable recovery time. With REBOA, aortic blood flow can be controlled via the insertion of a balloon-tipped catheter inside the aorta through the common femoral artery. REBOA allows for hemorrhage control via cessation of aortic flow across the balloon thereby promoting clot formation and hemodynamic stabilization. REBOA also augments blood flow to organs cranial to the point of occlusion, including the brain and heart. The development of low-profile REBOA catheters (11) as well as recent translational and clinical research have contributed to significant advancements in REBOA implementation. REBOA was originally developed for the management of patients with non-compressible torso hemorrhage (NCTH) due to trauma and has since found applications in other fields, such as post-partum and elective surgery hemorrhage control. There is also a growing body of research evaluating the use of REBOA for cardiopulmonary resuscitation (CPR). This review will describe the implementation of several REBOA strategies (complete, intermittent, and partial occlusion), discuss its clinical benefits and limitations, and describe possible applications in veterinary medicine.

## REBOA Implementation

### Catheter Description

**Figure 1:**
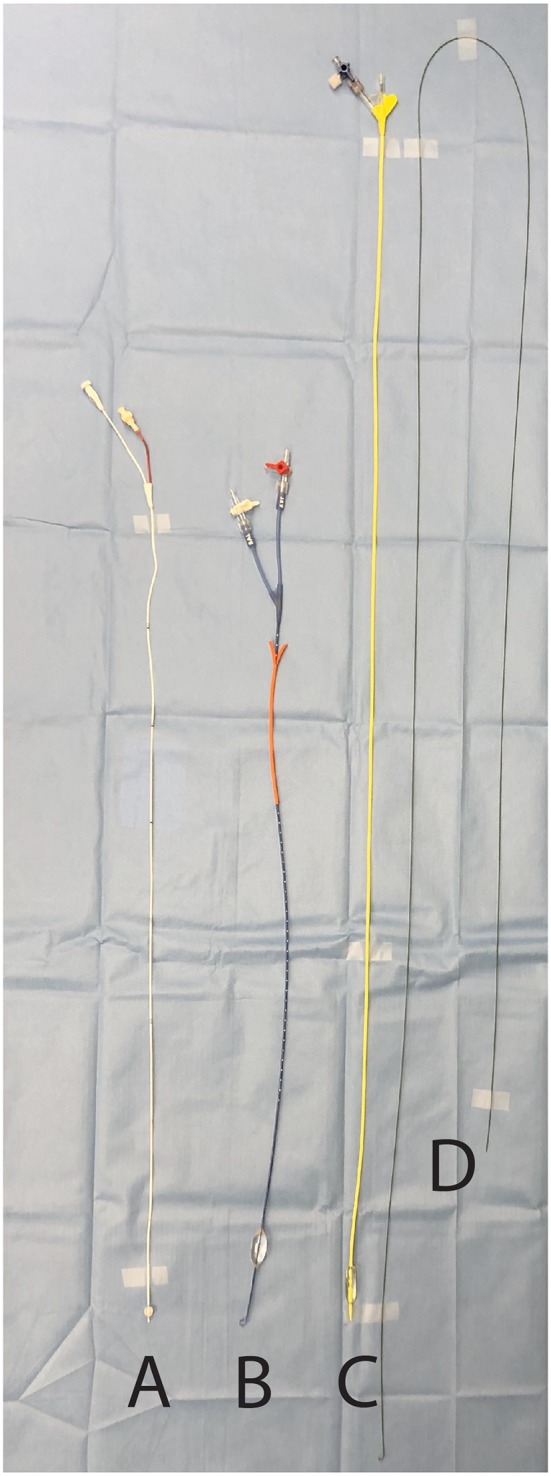
Examples of balloon-tipped catheters used for resuscitative endovascular balloon occlusion of the aorta. **(A)** Fogarty® Occlusion Catheter (Edwards Lifesciences, Irvine, CA) **(B)** ER-REBOA® (Prytime Medical, Boerne, TX) **(C)** CODA-LP® balloon catheter (Cook Medical, Bloomington, IN) **(D)** 0.035″ Guidewire (Terumo, Tokyo, Japan) required for the placement of **(C)**.

There are currently several commercially available catheters commonly used for REBOA in clinical practice in human patients (12). The first is the CODA balloon catheter (Cook Medical, Bloomington, IN), which is an over-the-wire 12 Fr device inserted through a 12 Fr introducer sheath with a balloon volume of 60 mL. The second is the CODA-LP® balloon catheter (Cook Medical, Bloomington, IN), which is a 9 Fr device inserted through a 9 Fr introducer sheath. It is also an over-the-wire catheter and has a balloon volume of 30 mL. The third catheter is the ER-REBOA® (Prytime Medical, Boerne, TX), which is a 7 Fr catheter that does not require a guide wire for placement and can be inserted through a 7 Fr introducer sheath (Figure 1). It has a balloon volume of 24 mL and has a cranial port for arterial sampling or blood pressure transduction. The ER-REBOA® is currently the only commercially available device specifically designed for REBOA. A newer catheter (pREBOA-PRO, Prytime Medical, Boerne, TX) developed for the purpose of partial aortic occlusion has recently been used in swine and is not yet commercially available (13, 14). Other devices include the Rescue Balloon Occlusion Catheter (7 Fr sheath, 40 mm balloon, Tokai Medical Products, Kasugai, Japan), the RELIANT® Stent Graft Balloon Catheter (12 Fr sheath, 10–46 mm balloon, Medtronic, Dublin, Ireland), the Fogarty® Occlusion Catheter (5 Fr sheath, 11 mm balloon volume, Edwards Lifesciences, Irvine, CA) (Figure 1), the Q50® PLUS Stent Graft Balloon Catheter (12 Fr sheath, 10–50 mm balloon) and ResQ™ Occlusion Balloon Catheter (11 Fr sheath, 10–38 mm balloon) (Qx Médical, Roseville, MN). Regardless of the device used, the REBOA balloon is made of a compliant material and inflated with 0.9% sodium chloride.

### Catheter Placement and Occlusion Zone Selection

**Figure 2:**
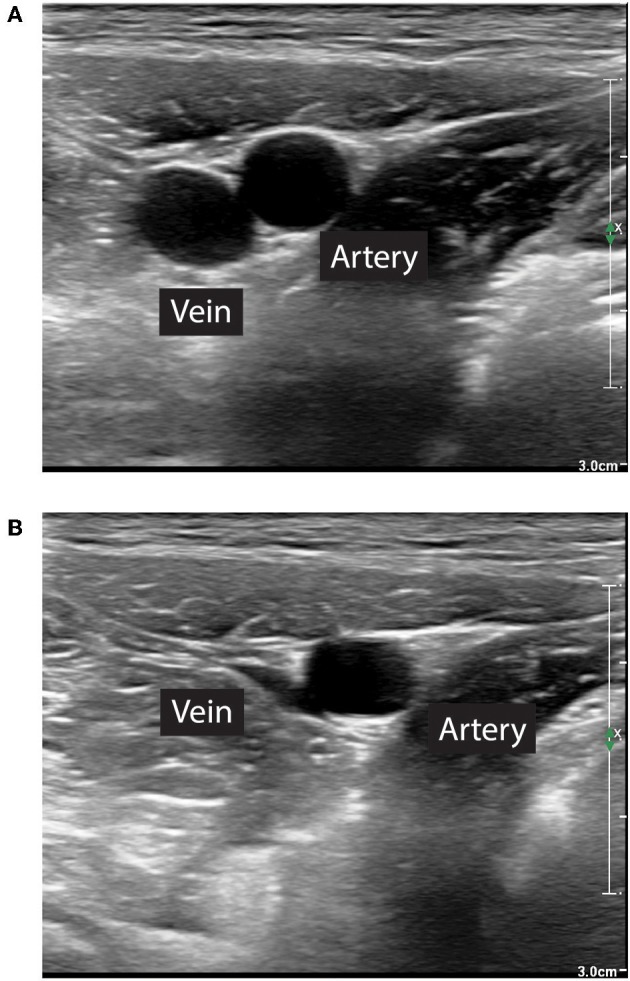
Differentiation of femoral artery and vein for ultrasound guided transcutaneous placement of the introducer sheath. **(A)** The femoral artery is smaller, pulsatile. **(B)** The femoral artery diameter does not decrease with light compression with the ultrasound probe.

**Figure 3:**
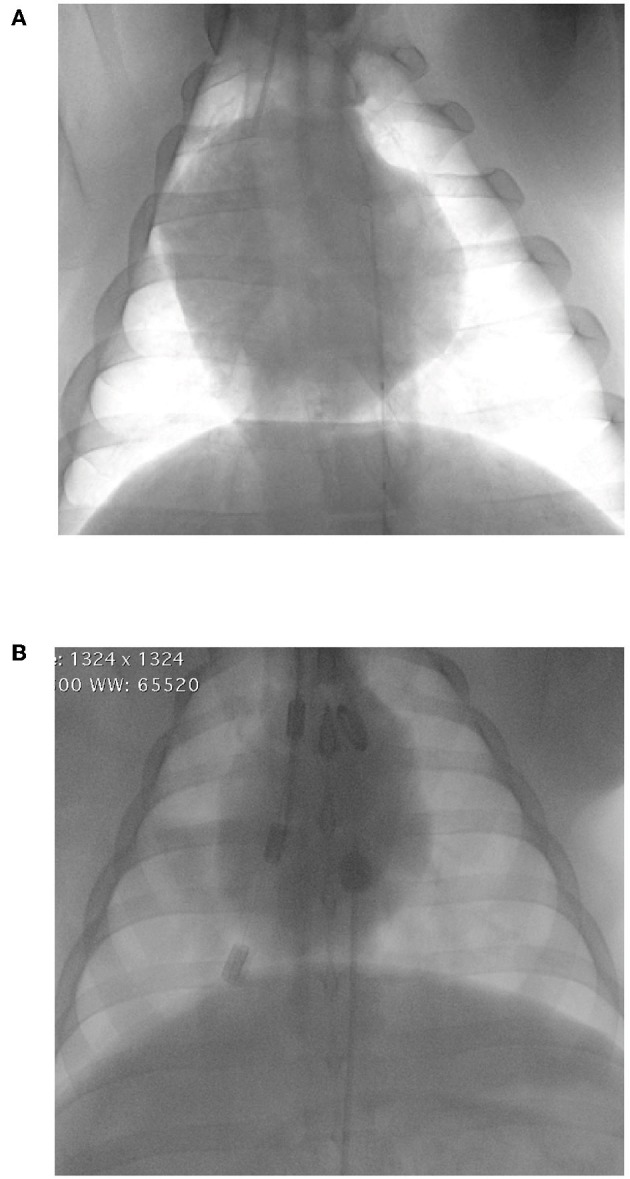
Fluoroscopic images of the proper placement of resuscitative endovascular balloon occlusion of the aorta (REBOA) catheters in zone 1 (supra-celiac location). **(A)** ER-REBOA® (Prytime Medical, Boerne, TX) **(B)** Fogarty® Occlusion Catheter (Edwards Lifesciences, Irvine, CA).

While the technique has been described in humans and several animal models, there is no description of the clinical use of REBOA in dogs or cats. The following is therefore derived from human clinical practice. Femoral arterial access can be achieved by either pulse palpation or using anatomical landmarks. Since patients with severe hemorrhagic shock may present with weak or absent pulses, ultrasonographic guidance is a popular tool to facilitate prompt catheter insertion in humans. The use of ultrasound also facilitates vessel evaluation to avoid venous catheterization or observe puncture-induced complications, such as hematomas or vessel wall dissection. The femoral artery can be differentiated from the femoral vein on ultrasound by its pulsatility, smaller diameter, and resistance to compression with the ultrasound probe (Figure 2). Using the Seldinger technique, an introducer sheath is placed in the femoral artery. The balloon at the tip of the REBOA catheter can be placed at various levels, or zones, of the aorta. Zone 1 encompasses the segment of the aorta between the left subclavian artery and the celiac trunk, zone 2 extends from the celiac trunk to the most caudal renal artery, and zone 3 is between the most caudal renal artery and the aortic bifurcation. Thus, in patients with profound hemorrhagic shock due to abdominal trauma and abdominal or pelvic hemorrhage, if the source of bleeding is unknown, the balloon can be placed in zone 1. If the lesion is deemed infrarenal, such as in a patient with significant pelvic fractures, the balloon can be placed in zone 3. Zone 2 placement is seldom performed. Proper balloon placement can be verified either via fluoroscopy (15) (Figures 3A,B), ultrasound (16), radiographs, or manual palpation during celiotomy. Measurement of catheter length of insertion using anatomical landmarks has been described in both humans (17–19) and dogs (20), which provides insight to facilitate translation into veterinary clinical practice.

### Occlusion Zone and Duration

The total tolerable duration of occlusion is dependent upon the zone of placement. For patients with an injury caudal to the celiac artery and cranial to the renal arteries, zone 1 occlusion is recommended; for those with an injury caudal to the renal arteries, zone 3 occlusion can be implemented. Zone 1 occlusion is currently limited to 30–45 min (21) while occlusion in zone 3 has been carried out for up to 60 min. The application of external limb cooling in pigs undergoing zone 3 occlusion following hemorrhagic shock for 6 h reduced damage to limb muscles as evidenced by reduced circulating creatine kinase activity (22), but a lack of functional testing leaves this method highly controversial. To leverage the advantages of zone 1 and zone 3 occlusion, a novel REBOA algorithm has been proposed (23): in hypotensive (systolic blood pressure <90 mmHg) trauma patients, if damage to the thoracic aorta is ruled out (radiographs or lack of effusion on thoracic ultrasound), the REBOA balloon can be inflated in zone 1. If there is no favorable response, emergency laparotomy should be promptly performed to control the source of bleeding. If the patient's blood pressure improves and the patient has hemorrhagic peritoneal effusion, laparotomy can be delayed as medical resuscitation efforts (blood products, fluids, and/or vasopressors) continue. In those patients who respond to zone 1 occlusion without peritoneal effusion, pelvic radiographs should be acquired. In case of pelvic fractures, the balloon can be moved from zone 1 and inflated in zone 3 to reduce ischemia to abdominal organs. If no pelvic fracture is observed, it is possible the patient's hemorrhage responded to medical resuscitation and zone 1 occlusion allowed for clot formation and stabilization.

### Balloon Inflation

Following catheter placement, the balloon is inflated using 0.9% sodium chloride. Initially the balloon should be inflated until complete aortic occlusion, a practice termed complete REBOA. Complete occlusion can be confirmed by the absence of pulse caudal to the balloon either by palpation or waveform observation via arterial pressure transduction from the femoral sheath. Full aortic occlusion allows for hemorrhage cessation, clot formation, as well as augmentation of perfusion to organs cranial to the balloon (heart and brain). Complete REBOA is nonetheless associated with a significant ischemic burden caudal to the balloon. Current REBOA guidelines recommend a maximum complete occlusion time of 30–45 min in zone 1, consistent with observations from retrospective studies (8). Longer occlusion times often result in serious ischemic injuries (24).

### Balloon Management During Inflation

To reduce the ischemic burden, clinical and translational research has focused on partial REBOA, whereby, following a brief period of complete REBOA (~10–15 min), a small amount of blood flow is allowed around the catheter to maintain oxygen delivery to tissue beds caudal to the balloon and reduce the ischemia-reperfusion burden observed prior to- and after balloon deflation. According to a recent position statement, the term partial REBOA should be used “*to describe the general approach of partial balloon catheter inflation for the purpose of resuscitating the physiologically deranged patient, with the dual goal of minimizing downstream ischemic injury while limiting hemorrhage*” (25). There is currently no catheter fitted with flow-measuring capabilities, therefore most approaches are based either on pulse palpation or cranial/caudal arterial pressure evaluation. Partial occlusion has been controlled by deflating the REBOA balloon until return of pulses caudal to the balloon or pulsation on the caudal aortic waveform (26). Precise aortic flow titration is important to sustain caudal perfusion while reducing the risk of cardiovascular collapse. Swine studies have shown that allowing the caudal mean arterial pressure (MAP) to rise by 10 mmHg above its value during complete occlusion correlates with an aortic flow of 250–500 mL/min depending on the severity of shock (27). For instance, if the caudal MAP during complete occlusion was 15 mmHg, aortic flow should be re-instituted by deflating the balloon until the caudal MAP reaches 25 mmHg. Other studies have suggested titration of aortic flow based on cranial systolic pressure (28) or measurement of aortic diameter (29). The use of precision syringes (Encore 26 Advantage Kit, Boston Scientific Corporation, Marlborough, MA) might facilitate titration of blood flow. Development of an automated platform capable of precise titration of balloon volume (on the microliter scale every few seconds) to control aortic blood flow as well as cranial and caudal MAPs is underway (30, 31). This dynamic, automated, and precise control of endovascular aortic balloon has been termed endovascular variable aortic control (EVAC) (31).

An alternative to allowing a partial flow past the balloon is to intermittently deflate the balloon completely and then re-inflate the balloon to allow a short period of perfusion; a practice termed intermittent REBOA (32, 33). Intermittent REBOA has been described in swine and has been implemented either in a time- or pressure-based fashion. With the time-based approach the balloon is deflated at pre-set time intervals and then re-inflated. The pressure-based approach mandates balloon inflation when the MAP falls below 30–40 mmHg (32, 33). This approach has not yet been directly compared to a partial REBOA technique and there is a paucity of clinical research to understand how much additional time intermittent REBOA provides the user.

### Resuscitation and Hemorrhage Source Control

Deployment of a REBOA catheter is only a bridging intervention that allows rapid control of hemorrhagic shock. Once the hemorrhage is controlled via balloon inflation and the patient is stabilized, further resuscitation efforts, such as blood products, crystalloids, and vasopressor administration can be initiated. Abdominal imaging should be performed to assess the extent of injury. Contrast injection through the REBOA catheter can also be used to facilitate computed tomography-based lesion diagnosis (13). Furthermore, emergency damage control surgery or endovascular interventions is required for definitive hemorrhage control.

### Balloon Deflation and Continued Resuscitation

After fluid resuscitation and definitive hemorrhage control, via surgery or interventional radiology, the intra-aortic balloon can be deflated, which is associated with significant reperfusion injury. Balloon deflation is therefore carefully controlled to prevent cardiovascular collapse. The balloon is usually deflated incrementally over 10 min, but deflation time should be adjusted to occlusion time. Shorter deflation times may be better tolerated in shorter occlusion times. Manual balloon deflation may lead to unpredictable changes in aortic flow and MAP. In one swine study, removal of 0.5 mL of 0.9% sodium chloride from the REBOA catheter balloon every 30 s yielded a wide range of changes in aortic flow and MAP. Furthermore, initial return of aortic flow was unpredictable (34). Hypotension because of balloon deflation is a common problem in translational research as well as clinical practice in human patients. The balloon can be progressively deflated in a controlled and dynamic fashion to control reperfusion-induced hypotension. This novel approach has been termed endovascular perfusion augmentation for critical care (EPACC) (30). When EPACC is implemented, the balloon is slowly deflated by an automated platform. If the patient becomes hypotensive (usually with a MAP <65 mmHg), the balloon is re-inflated until the cranial MAP reaches a preset target (usually 65 mmHg). Patients can then benefit from fluid and vasopressor resuscitation in conjunction with cranial MAP augmentation via balloon inflation. The automated platform makes minute changes to balloon volumes to maintain blood pressure within target range. When compared to complete REBOA, partial REBOA and EVAC were associated with reduced ischemia-reperfusion burden at the time of balloon deflation as evidenced by reduced plasma lactate concentration as well as lower isotonic crystalloids and vasopressor requirements (31, 35).

## Additional REBOA Challenges

Complications associated with the use of REBOA catheters are either related to the technique itself or are consequences of ischemia-reperfusion injury (Table 1) (36).

**Table 1:** Technical and metabolic complications associated with resuscitative endovascular balloon occlusion of the aorta (REBOA) (11, 21, 24, 36–41).

	**Technical complications**	**Metabolic complications**
Introducer sheath complication	Wrong vessel cannulation Vessel injury Air embolus Distal ischemia Hemorrhage Thrombosis	Acute kidney injury Liver failure Spinal cord infarction Intestinal ischemia Myonecrosis Limb loss Hyperkalemia Metabolic acidosis Thrombosis Hypotension Myocardial damage Ongoing hemorrhage upon balloon deflation Death
Improper balloon placement	Improper deployment location - Failure to stop hemorrhage - Arterial damage if balloon deployed in a small artery (celiac, renal, or mesenteric) Balloon migration	
Balloon over inflation	Balloon rupture Vessel dissection Vessel rupture	
Sheath removal complication	Hemorrhage Thrombosis Arterial dissection Limb ischemia Vasospasm	

### Technical Complications

Development of low-profile devices has improved the safety of REBOA. The use of smaller catheters, inserted through a 7 Fr sheath rather than 11–12 Fr sheath, has been associated with reduced need for cut-down vascular access, distal extremity embolism, as well as mortality (42). The introducer sheath may be inadvertently placed in a femoral vein instead of an artery. Proper placement can be ensured by identification of the femoral artery over the femoral vein (size, anatomical landmarks, pulsatility, compressibility, Figure 2), pressure transduction through the sheath, or blood gas analysis using a sample from the sheath. REBOA catheter deployment can be further complicated by vessel injury and hematoma at the time of insertion or upon removal of the insertion sheath. Despite proper positioning, the balloon can be displaced (37).

### Metabolic Complications

A list of common metabolic complications of REBOA is presented in Table 1. Trauma patients are often presented with a wide range of tissue injuries. These direct injuries to tissues can be worsened by ischemia from hemorrhagic shock, and, when REBOA is used, the substantial resulting ischemia-reperfusion injury. While there is a paucity of mechanistic data, ischemia-reperfusion injury is often reported in animal models of REBOA. Commonly used markers of ischemia-reperfusion injury include plasma pH, and base excess, along with plasma lactate and potassium concentrations (23, 43–46). Such observations have been confirmed via intraperitoneal microdialysis measuring lactate and pyruvate concentrations (47).

Injuries to the kidneys, liver (32, 38, 45, 48), gastrointestinal tract (38, 49), and spine (45, 49, 50) are often reported. Patients with NCTH treated with REBOA are at risk for acute kidney injury (AKI). In those patients, AKI is likely multifactorial. According to a recent systematic review (51), renal damage was reported in 3/3 human trauma studies where REBOA was employed (39, 52, 53). Interestingly, only 1/3 of those studies utilized zone 1 occlusion (39) (1/3 study did not report the point of occlusion), suggesting that even infrarenal aortic occlusion is associated with AKI. Similar observations have often been reported in animal studies (32, 38, 44, 45, 48, 49, 54). Hyperkalemia has also often been observed following REBOA (44, 46), which most likely results from potassium release from damaged cells and decreased renal excretion. Careful monitoring of electrolytes, specifically potassium and calcium concentrations, and prompt treatment of imbalances are therefore important following reperfusion.

Supraphysiologic arterial blood pressure cranial to the point of occlusion during REBOA has raised concerns regarding injury to organs cranial to the balloon. REBOA use is contraindicated in patients with hemorrhage cranial to the left subclavian artery for fear of exacerbating cranial thoracic hemorrhage. Myocardial injury following REBOA has been reported. It has been attributed to increased afterload leading to increased myocardial strain in conjunction with reperfusion injury following aortic occlusion. Various swine models of hemorrhagic shock have demonstrated elevation in circulating levels of troponin along with histopathologic evidence of myocardial injury (32, 40, 44). Early clinical data raised concerns for exacerbation of intracranial hemorrhage in patients with NCTH and traumatic brain injury due to arterial blood pressure augmentation (55). However, translational research has shown that REBOA, especially partial REBOA strategies, do not worsen intracranial hemorrhage associated with traumatic brain injury, although larger human observational data is needed (14, 35).

## Current Indications for REBOA

### Non-compressible Torso Hemorrhage

NCTH is currently the leading indication for REBOA. The term NCTH has long been used in the literature to refer to hemorrhage from a location where no point of compression could be applied to control bleeding (56). According to a more recent definition (57), NCTH is recognized in patients with vascular damage from one or more of the following: thoracic trauma, ≥grade 4 solid organ (liver, kidney, or spleen) injury, major torso vessel injury, or pelvic fracture with ring disruption. Additionally, these patients must present with hemorrhagic shock (systolic arterial pressure <90 mmHg) or demonstrate the need for immediate operation. Establishing a definition for NCTH has allowed for improvement in the quality of the evidence via enrollment of a homogenous population of patients with similar conditions. In particular, this definition has permitted separation of patients with NCTH from those with non-compressible truncal injury (NCTI), which is a population with injuries placing them at risk for NCTH without the presence of hemorrhagic shock.

Much of the original REBOA data stemmed from military research. Data from the Joint Theater Trauma Registry (JTTR) established that 68% of battlefield-acquired wounds were penetrating, which is much higher than in the civilian population (11%) (58). Novel personal protective equipment for the war-fighter along with new paradigms of prehospital care (58), and improvement of en-route care from point of injury to a medical facility (59) have allowed a decrease in morbidity and mortality due to battlefield-acquired wounds (60). Additionally, the increased use of tourniquets (60, 61) and hemostatic materials led to a reduction in deaths due to extremity hemorrhage; furthermore, body armor use has provided increased protection to the war fighter's thorax. These advances have placed abdominal and junctional hemorrhage as major causes of morbidity and mortality. Recent studies categorizing patient death as retrospectively *survivable, potentially survivable*, or *preventable deaths*, have led to resurgence in NCTH treatment research. A review of special operation forces causes of death between 2001 and 2004 showed that of the 82 fatalities, 15% (12/82) had potentially survivable injuries. NCTH was listed as a cause of death in 50% of those patients (56). Similar results have shown that NCTH is a significant source of preventable death in other cohorts (62, 63). NCTH is also a significant problem in the civilian population, especially in scenarios of natural or man-made disasters. A study examining the National Trauma Data Bank, which included 1.8 million patients between 2007 and 2009, reported that of 249,505 patients with NCTI, 20,414 (8.2%) had NCTH, with an associated mortality rate of 7 and 45%, respectively (64).

**Figure 4:**
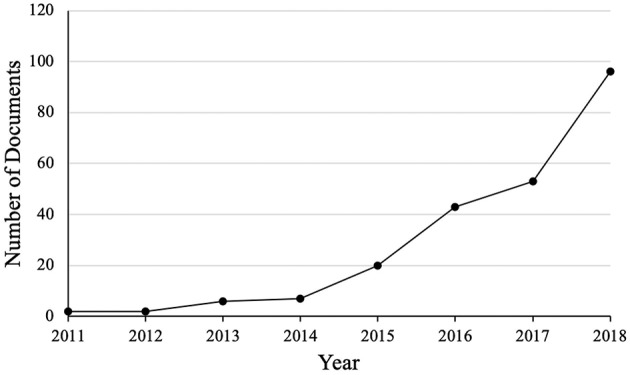
Number of documents listed in the Scopus database between 2011 and 2018. Documents include articles (114), reviews (32), conference papers (31), letters (18), articles in press (11), notes (8), editorials (5), erratums (5), short surveys (3), book chapter (2) (total = 229). Search terms: REBOA or resuscitative endovascular balloon occlusion of the aorta (Source: Scopus, accessed 03/01/2019).

Due to the potential benefits of a minimally-invasive solution for NCTH management, there has been a surge in publications related to REBOA over the recent years (Figure 4). Additionally, the recent production of a 7 Fr REBOA catheter (ER-REBOA®, Prytime Medical, Boerne, TX) may have contributed to the rising interest in REBOA since a 7 Fr access sheath does not require arterial wall closure in humans. Finally, REBOA appears safer for providers than RT and has increased application in out-of-hospital settings. REBOA has now been utilized in austere environments, in both civilian (65, 66) and military (67) theaters.

## Expanded Uses of REBOA

### Intraoperative Use in Non-trauma Settings

REBOA has been used as an adjunct in many non-traumatic surgical settings commonly associated with profound blood loss, for instance: hemorrhage control in patients with major gastrointestinal hemorrhage (41, 68, 69), abdominal aortic aneurysm rupture (41, 68, 69), or those undergoing complex tumor removal (70, 71). REBOA has also been used in women with abnormal placentation, who are at risk for life-threatening hemorrhage (68, 72). In one case report, REBOA therapy allowed for hemorrhage control and successful cesarean hysterectomy in a Jehovah's witness with placenta percreta, a procedure usually lethal in this patient population (73). Such intraoperative use could potentially be considered in veterinary patients of adequate size.

### Cardiopulmonary Resuscitation

A REBOA catheter can be placed in patients with traumatic (66, 74) or non-traumatic cardiopulmonary arrest (CPA) (75–77). Aortic occlusion during CPR allows for selective perfusion to the myocardium and brain. A swine study of non-traumatic CPA showed that aortic occlusion at the level of the diaphragm resulted in increased mean and diastolic arterial pressure, which increased coronary perfusion pressure. Furthermore, aortic occlusion also resulted in improved markers of ischemic injury (higher pH and lower plasma lactate concentrations) when compared to controls or those undergoing aortic occlusion at the level of the heart (28). Similar animal studies showed improvement in myocardial perfusion pressure with the use of REBOA during CPR (78–80). Arterial pressure augmentation with balloon occlusion of the aorta has also been described in human patients undergoing CPR (81). One study investigated the use of intra-aortic vasopressin administration in combination with balloon occlusion of the aorta (82) and showed that in addition to perfusion pressure augmentation, this approach may have beneficial effects on cerebral blood flow following return of spontaneous circulation. Norepinephrine did not have a significant benefit on cerebral perfusion pressure when delivered in a similar manner (83). A porcine survival study showed that intra-aortic administration of epinephrine in combination with aortic balloon occlusion did not improve outcome (84).

## Future of REBOA in Veterinary Medicine

### Catheter Size

**Figure 5:**
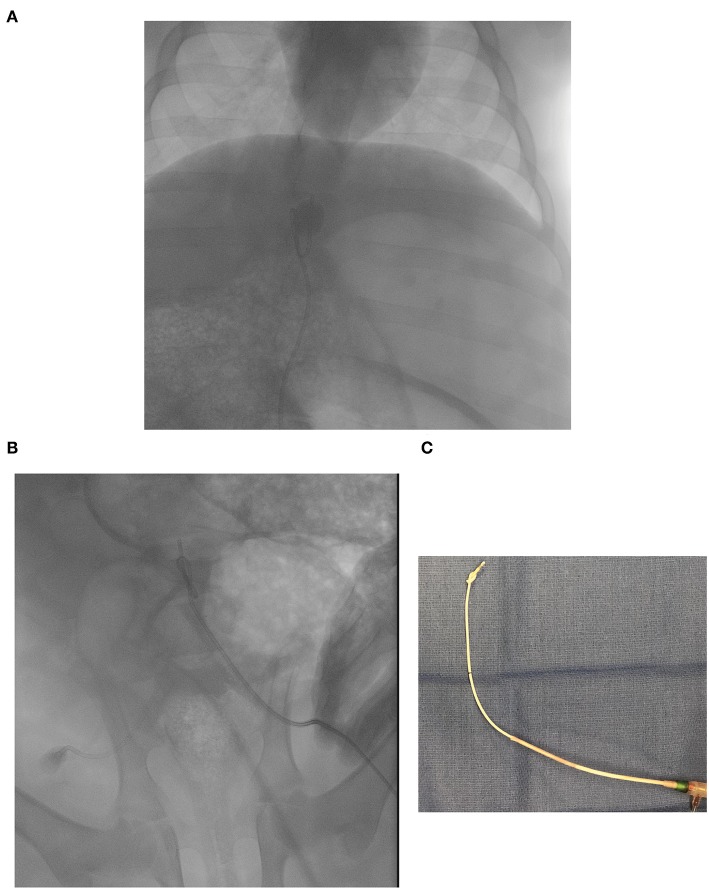
Resuscitative endovascular balloon occlusion of the aorta (REBOA) catheter balloon migration following complete aortic occlusion in zone 1 (supra-celiac location). **(A)** Fluoroscopic image of a caudally displaced endo-aortic balloon. **(B)** Fluoroscopic image of a balloon that tied in a knot during occlusion. **(C)** Picture of the REBOA catheter tip in **(B)** after removal.

Aside from cost, catheter availability for veterinary patients is a significant limitation in the adoption of REBOA in clinical veterinary practice. While there are no guidelines in veterinary medicine, the use of currently available purpose-made REBOA catheters would be limited to canine patients of medium to large breeds. A similar problem is observed in human pediatric patients. Due to the size of their vessels (85), children under the age of 8 are too small to be fitted with a 7 Fr introducer sheath. Non-REBOA specific catheters have been repurposed to perform REBOA in pediatric-size swine (Figure 3B, data not published), but those catheters are not stiff enough and the balloon sometimes gets displaced (Figure 5A) or tied into a knot (Figures 5B,C).

### Translational Medicine Contribution

A significant body of the REBOA literature has been built on animal studies. While the pig has been the leading species (86), there are also several ovine reports of REBOA (29, 46), although less common. It is important to note that the dog has been used as a model for REBOA research especially during CPR, which might facilitate its translation into veterinary clinical practice. REBOA has been used in a non-traumatic canine model of hemorrhagic shock (87). Following 34 min of hemorrhage to a MAP of ~50 mmHg, animals who were treated with aortic occlusion and a bolus of hypertonic sodium chloride or sodium acetate displayed improved cardiovascular function when compared to those who receive aortic occlusion and isotonic sodium chloride, with transient increases in markers of cardiac function (cardiac index, systolic index, and cardiac filling pressure). The rest of the canine literature in canine endovascular balloon occlusion stems from CPR research. In a canine study of ventricular fibrillation-induced CPA, zone 1 occlusion resulted in improved myocardial perfusion pressure and more frequent return of spontaneous circulation. In this study the balloon was inflated 3 min after CPR initiation and deflated over 2 min in those animals that sustained ROSC for at least 6 min (88). In open chest CPR studies, dogs that underwent endovascular balloon aortic occlusion displayed higher myocardial (89, 90) and cerebral (89–91) blood flow when compared to controls. The use of endovascular aortic occlusion in CPA has led to the development of selective aortic arch perfusion, whereby resuscitation drugs, including fluids, are infused through the aortic balloon catheter. Canine studies have established selective aortic arch perfusion benefits on myocardial perfusion pressure in ventricular fibrillation-induced CPA (92–94).

### Clinical Use for Veterinary Medicine

Veterinary patients may benefit from REBOA, especially if smaller and affordable devices are developed. One study showed that the balloon of a REBOA catheter was successfully placed in 15/15 canine cadavers using the 12th thoracic vertebrae as an external landmark (20). The device used in this study utilized a REBOA catheter requiring a 7 Fr introducer sheath. Femoral artery access was established via either cut-down or ultrasound guidance, and the catheter placed in dogs with weight ranging from 10 to 48 kg. While balloons were inflated with a median of 0.4 mL/kg (range, 0.21–0.67) of iohexol solution, we caution practitioners about the use of balloon volume as a target for occlusion as over inflation is a significant concern with this approach. Complications (linear defect and focal aortic dissection) were observed in two of the five cadavers that underwent histopathology (20).

We have used the Fogarty® Occlusion Catheter in piglets weighing ~20–30 kg (unpublished data). While we have been successful at achieving complete aortic occlusion in hemorrhagic shock models, caudal migration of the balloon is not uncommon (Figure 5A). We also observed some catheter tips being tied in a knot (Figures 5B,C). This catheter is not inserted over a wire and may not be stiff enough to withstand supraphysiologic MAP.

There are ample possibilities for the use of REBOA in veterinary patients, provided the availability of a catheter that can be safely inserted in smaller patients. In addition to traumatic hemorrhagic shock, REBOA could be used in several scenarios in veterinary patients, for instance: unstable patients with non-traumatic hemoperitoneum, complex tumor resection, or salvage from intra-operative iatrogenic injury. As an example, we have observed one instance of iatrogenic aortic laceration during fenestration of an intervertebral disc for which aortic occlusion would have facilitated patient stabilization and aortic repair.

Since management of patients undergoing REBOA requires advanced care during and after the procedure, proper training is important. Similar to human medicine providers, veterinarians are encouraged to gain experience in advanced vascular access and management of complications from endovascular interventions. In humans, the Basic Endovascular Skills for Trauma (BEST) course from the American College of Surgeon provides core information for safe REBOA implementation. Complex balloon inflation/deflation cycles might be facilitated by artificial intelligence algorithms specifically tailored to the pathophysiologic specificities of veterinary patients undergoing REBOA. Clinicians should keep in mind concerns for complications following introducer sheath removal (massive hemorrhage, vasospasm or thrombosis leading to caudal ischemia) as arterial wall repair is not commonly done in clinical practice in veterinary medicine and may require advanced training. True indications for placement of a REBOA catheters remain debated in the trauma literature (95, 96). Adequate patient selection as well as involvement of trained staff in the appropriate environment seem to be important (95, 96).

## Conclusion

The practice of REBOA has benefited from significant advances mostly aimed at reducing ischemia-reperfusion injury. Animal research, in both traumatic and non-traumatic diseases, have yielded important information to this growing field, gaining more and more applications. While there is no published study reporting its clinical use in veterinary patients to date, REBOA might have application in veterinary practice. Training in all steps of the procedure is important, and a multidisciplinary approach involving emergency, critical care, anesthesia, and surgery personnel is likely to improve patient outcomes.

## Author Contributions

GH, ET, CB, MS, and ED conducted the literature review. GH and ET drafted the manuscript. CB, MS, ED, EF, LN, JG, IS, TW, and MJ edited the manuscript. ET, CB, MS, ED, EF, LN, JG, IS, TW, and MJ provided details regarding the practical use of REBOA in humans and pigs.

### Conflict of Interest Statement

MJ, LN, and TW are the founders of Certus Critical Care, which engages in the development of REBOA catheters. The remaining authors declare that the research was conducted in the absence of any commercial or financial relationships that could be construed as a potential conflict of interest.

## Abbreviations

AKI: acute kidney injury
CPA: cardiopulmonary arrest
CPR: cardiopulmonary resuscitation
EVAC: endovascular variable aortic control
EPACC: endovascular perfusion augmentation for critical care
MAP: mean arterial pressure
NCTH: non-compressible torso hemorrhage
NCTI: non-compressible torso injury
REBOA: resuscitative endovascular balloon occlusion of the aorta
RT: resuscitative thoracotomy.
